# Merkel Cell Carcinoma: The Past, the Present, and the Future

**DOI:** 10.1155/2013/929364

**Published:** 2013-04-16

**Authors:** Inamaria Erovic, Boban M. Erovic

**Affiliations:** ^1^Medical University of Vienna, 1090 Vienna, Austria; ^2^Medical University of Vienna, Department of Otolaryngology, Head and Neck Surgery, 1090 Vienna, Austria

## Abstract

Since the first description of the Merkel cell carcinoma by Cyril Toker in 1972, the number of studies has significantly increased over the last 4 decades. In this review, we will illustrate the historical background of the Merkel cell carcinoma beginning with the 19th century, the first description of the Merkel cell to the finding of the CK20 as a highly specific diagnostic marker and finally to the recently detected Merkel cell polyomavirus (MCPyV). Moreover, we will highlight the beginning of adjuvant therapeutic regimens with radiotherapy and chemotherapy and discuss the diagnostic work-up including imaging and histology of patients with Merkel cell carcinoma. Another very rapidly growing and interesting field of research is the development of patients' specific and tailored targeted therapy, in particular in patients with distant metastatic disease.

## 1. Introduction

Since the first description of the Merkel cell carcinoma by Cyril Toker in 1972, the number of studies has significantly increased over the last 4 decades. In this review, we will illustrate the historical background of the Merkel cell carcinoma beginning with the 19th century, the first description of the Merkel cell to the finding of the CK20 as a highly specific diagnostic marker and finally to the recently detected Merkel cell polyomavirus (MCPyV).

Moreover, we will highlight the beginning of adjuvant therapeutic regimens with radiotherapy and chemotherapy and discuss the diagnostic work-up including imaging and histology of patients with Merkel cell carcinoma.

Another very rapidly growing and interesting field of research is the development of patients'-specific and tailored targeted therapy, in particular in patients with distant metastatic disease.

## 2. Finding the Merkel Cell

**Figure 1:**
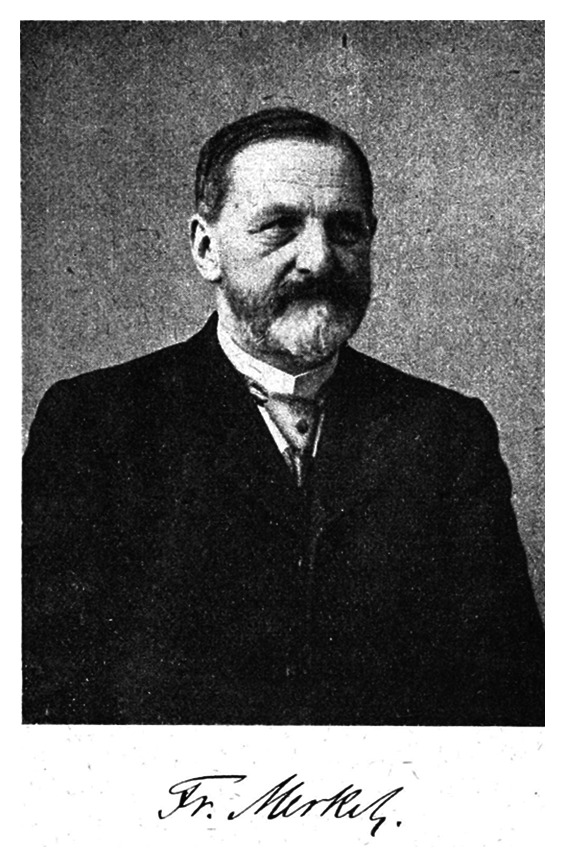
Friedrich Sigmund Merkel (1845–1919).

Friedrich Sigmund Merkel was born on April 5, 1845 and died on May 28, 1919. He was a German anatomist and histopathologist who first described the so-called *Tastzellen* or touch cells in the skin [1] (Figure 1). Interestingly, three years later the term Merkel cell was born, by a young anatomist Robert Bonnet (1851–1921) who later worked with Dr. Merkel. 

In mammals, Merkel cells are localized in the basal layer of the skin and mucosa [2] either as single cells or in clusters (in german *haarscheiben*). Clusters contain about 50 cells (touch domes) and are in close neighborhood to nerve terminals forming mechanoreceptors [2]. There are other cells called “Merkel-cell-like” cells, also in the skin and mucosa but without contact with nerve terminals. They are probably part of a diffuse neuroendocrine system and do not function as mechanoreceptors. Probably, these cells, rather than those acting as mechanoreceptors, are the origin of the highly malignant Merkel cell carcinoma. Recent studies could show that Merkel cells originate from the neural crest [3] and are found in the skin and parts of the mucosa that are derived from the ectoderm. 

### 2.1. Structure of Merkel Cells

With the introduction of the electron microscopy in medicine in the 1960s, new significant knowledge in regard to cellular anatomy was gained. Particularly, in 1965 and 1969 Munger, Iggo and Muir showed that Merkel cells are clear and oval cells, measuring approximately 10–15 *μ*m in the long axis having lobulated nuclei that contain intermediate cytokeratin and neurofilaments [4, 5]. Moreover, Merkel cells have spike-like protrusions that enable them to interdigitate with the surrounding keratinocytes. The nerve terminals of the Merkel cells are packed with mitochondria and optically clear vesicles [4].

### 2.2. Protein Expression in Merkel Cells

The most interesting fact in regard to the protein expression profile is that epithelial proteins like cytokeratins but also neuroendocrine markers like neuron-specific enolase can be found in Merkel cells [2]. In particular, cytokeratin 20 is of significant value as a highly specific marker for Merkel cells in normal squamous epithelium [6]. Besides neuron-specific enolase, protein gene product 9.5, synaptophysin, and chromogranin A are found immunohistochemically in Merkel cells as well [2]. 

### 2.3. First Description of the Merkel Cell Carcinoma

In 1972, Toker first described a trabecular carcinoma of the skin [7]. In those days, he was a pathologist at the Mount Sinai School of Medicine, City University of New York and later Professor of Pathology and Head of the Division of Surgical Pathology at the University of Maryland Hospital and Medical School in Baltimore, MD, USA. He analyzed five cases and described clinical and histomorphological aspects. In particular, tumor cells displayed large, oval nuclei with vesicular chromatin and prominent nucleoli. The tumor growth pattern was trabecular and column-like infiltrating between dermal bundles. In regard to the origin of the trabecular carcinoma, Dr. Toker hypothesized that the carcinoma cells derived from epithelial structures are capable of forming primitive sudoriferous structures, that is, early fetal sweat glands. 

Six years later, in 1978 Tang and Toker found dense-core granules in three of the original tumors by electron microscopy [8]. Merkel cells are the only cells in the skin that have dense-core granules. This fact led subsequently to the hypothesis that this trabecular skin carcinoma arises from Merkel cells. Further electron microscope studies showed that both Merkel cells and Merkel cell carcinoma cells have overlapping electron microscopic features. On the protein level, immunohistochemical expression of Cytokeratin 20 supports the hypothesis that the Merkel cell is the cellular origin of this aggressive skin tumor [6]. However, to date there is a controversy going on regarding the origin of the Merkel cell carcinoma. Some authors believe that the Merkel cell carcinoma derives from pluripotent stem cells from the skin. Our research group could show as well that Bmi-1, a stem cell marker, was homogenously and highly positive in all Merkel cell carcinoma samples [9]. Therefore, throughout the last decades Merkel cell carcinoma has been described under trabecular carcinoma of the skin, cutaneous neuroendocrine carcinoma, and Merkel cell carcinoma. The name Merkel cell carcinoma was first proposed by De Wolff-Peeters in 1980 and remains the most used and accepted term [10]. 

However, whether the Merkel cell carcinoma truly derives from the Merkel cell is still to date very controversially discussed. Without any doubt, more studies are needed to elucidate the origin of Merkel cell carcinoma because systemic therapy in patients with disseminated disease would probably have a significant higher impact on survival and disease-free rates due to modifications based on the origin of the cancer cells. 

### 2.4. CK20 as the Key Diagnostic Marker for Merkel Cell Carcinoma

In the decades following its initial discovery, reports on the pathogenesis, course, and treatment of Merkel cell carcinoma were scarce attributing to its rarity as a disease entity, lack of biomarkers for diagnosis, and nonunified staging classifications. In 1992, Dr. Moll and colleagues recognized that Cytokeratin 20 (CK20) expression was highly specific for Merkel cell carcinoma [6]. In this study, 15 specimens with Merkel cell carcinoma were tested for CK20 using the immunoblotting and immunohistochemistry technique. All cases for CK20 were significantly positive, and the authors proposed that this marker is highly specific for Merkel cell carcinoma. Moreover, CK20 helps to distinguish between Merkel cell and small-cell lung carcinoma cells since both tumors are morphologically similar [6]. 

In the following years, new studies showed that approximately 5% of all Merkel cell carcinoma specimens lack CK20 expression [11]. As a consequence, Jaeger showed in a recently published review that besides CK20 expression neuron-specific-enolase (NSE) and neurofilament protein (NFP) is specific for Merkel cell carcinoma [12]. Another very important tumor marker is thyroid transcription factor-1 (TTF-1). TTF-1 is a very reliable and accurate diagnostic marker for small-cell lung carcinoma but it is not expressed by Merkel cell carcinoma [13]. Other “negative” markers are leucocyte common antigen (LCA) and cytokeratin-7 (CK7) that are positive in lymphoma [14, 15] and small-cell carcinoma of the lung (SCLC), respectively [16]. Differentiating malignant melanoma and Merkel cell carcinoma is based on CK20 positivity in Merkel cell carcinoma but negativity for HBM45, NKI/C3, and S-100 [17].

## 3. Prognostic and Predictive Factors in Merkel Cell Carcinoma

In a recently published study, it could be shown that immunosupression and advanced-stage disease was a significant predictor for decreased survival in 240 patients with Merkel cell carcinoma [18]. Interestingly, tumor size had no impact on survival [18]. Touzé and colleagues found that high antibody titers of MCPyV were a significant predictor for progression-free survival [19]. Another study performed by Poulsen and colleagues showed that again stage was a significant prognostic factor for better survival but that intratumoral CD8+ lymphocyte invasion was shown to be a significant biomarker for improved survival in MCC patients as well [20]. This observation could be underlined by the study performed by Sihto et al. This study group could show that in 116 patients that besides intratumor infiltration with CD8+ cells high CD3+ tumor count has a significant impact on patients' overall survival [21].

Clinical factors like tumor thickness, size, sex, and age are shown to not be a reliable prognostic factor for overall and disease-free survival [18, 20, 22].

### 3.1. Finding of the Merkel Cell Carcinoma Polyomavirus

In 2008, Feng and coworkers found novel viral sequences in four Merkel cell carcinoma tumor tissues [23]. After sequence analysis, it could be shown that they encoded for a polyomavirus which was subsequently named as Merkel cell polyomavirus. Further studies showed a prevalence of 40% to 100% of the MCPyV in Merkel cell carcinoma specimens [24].

In particular, polyomaviruses encode for large and small T-antigens which bind to host proteins facilitating (i) viral replication and (ii) inactivation of tumor suppressor proteins p53 and pocket retinoblastoma (pRb). Feng and colleagues observed a monoclonal viral integration 5 out of 10 (50%) patient samples and interestingly primary and metastatic MCC tissues from the same patient showed an identical viral integration pattern, indicating that the integration of MCV preceded the metastatic spreading of the cancer [23].

The number of studies dealing with the MCPyV expression significantly increased over the last 3 years [11, 25]. In particular, in a large Australian cohort Paik and colleagues could show that the MCPyV large T protein was only detected in 7% of the specimens localized in the head and neck area and in 24% from other anatomic sites [11]. Since the expression of MCPyV large T-protein in Merkel cell carcinoma specimens in patients with less sun exposure is unknown, our group recently conducted a study and showed that MCPyV large T-protein was highly expressed in primary as well as metastatic lesions [25]. This observation is highly clinically relevant in two points: firstly MCPyV large T-protein can be easily and cost-effectively detected by CM2B4, a highly sensitive and specific mouse monoclonal antibody, in specimens that lack CK20 immunoreactivity.

Secondly, since the expression of MCPyV large T-protein is homogenously overexpressed in primary and more important in metastatic lymph nodes it can be used as a target protein for systemic therapy in patients with disseminated disease with very poor outcome [26, 27].

### 3.2. Management of Patients with Merkel Cell Carcinoma

#### 3.2.1. Surgery and Postoperative Radiotherapy

The first retrospective study in regard to treatment and management of Merkel cell carcinoma patients was conducted at the MD Anderson Cancer Center [28]. Between 1966 and 1983, 41 patients with Merkel cell carcinoma were treated. It could be shown that wide surgical resection of the primary lesion with neck dissection and adjuvant radiotherapy is the best treatment for controlling locoregional disease [28]. The first and still to date solemn prospective trial was performed in 2003 by the TASMAN group [29]. Interestingly, this study showed that adjuvant radiotherapy significantly prolonged locoregional disease-free survival whereas radiation had no impact on patients' overall survival [29].

#### 3.2.2. Mohs Surgery

Mohs micrographic surgery was introduced by Dr. Frederic Mohs in the 1930s and became over the decades a reliable technique for resection of cutaneous tumors particular at delicate sites. In case of Merkel cell carcinoma, only a few reports are available. A retrospective study conducted by Gollard and colleagues presented excellent results with no recurrence rate after 3 years. However, only 8 patients were included in this study. Another paper including 45 patients with Merkel cell carcinoma showed that Mohs surgery is a reliable and cost-effective technique [30]. The authors compared the outcome of two groups: one with Mohs surgery alone and one with adjuvant radiotherapy. In the first group only 1 (4%) marginal recurrence and 3 in transit-metastasis could be observed whereas in the second group none recurrent disease were observed in the radiation group. Nevertheless, in both groups, overall and disease-free survival were not significantly different between treatment groups. The authors conclude that radiotherapy is beside surgical resection a key factor for successful management of patients with Merkel cell carcinoma [30].

#### 3.2.3. Radiotherapy

Merkel cell carcinoma is a highly radiosensitive skin tumor [10]. Studies could show that adjuvant radiotherapy to the primary site and the nodal basins significantly improves locoregional control and overall survival [23, 24]. In patients where no surgical treatment, due to low medical performance, can be offered, primary treatment with radiation shows an excellent outcome and locoregional control rates [28, 29]. Controversies still exist regarding the treatment of the neck. The majority of the cancer centers worldwide prefer doing a selective a neck dissection with adjuvant radiotherapy [31]. However, numerous studies showed that radiotherapy alone to the neck has comparable locoregional control rates to surgery [32–34].

Since the discovery of the MCPyV, future studies are showing whether its expression is able to stratify patients either to primary radiotherapy or surgery plus adjuvant radiotherapy treatment. Such stratification has already taken place in squamous cell carcinoma of the oropharynx. In these patients, the human papilloma virus status decides whether patients will undergo primary radiotherapy or surgery with adjuvant radiotherapy [35]. 

#### 3.2.4. Chemotherapy

In the mid-eighties, several studies were conducted to evaluate the efficacy of chemotherapy in patients with disseminated Merkel cell carcinoma disease [31, 36]. 

For the first attempts to treat MCC metastases, regimens were chosen similar to those used for small-cell lung carcinomas because of its neuroendocrine differentiation and histopathologic features [31]. George and colleagues introduced carboplatin and reported a positive effect on patients' progression-free survival [31]. In the following years, a huge number of case series were published presenting therapeutic outcome after single or combined treatment with radiotherapy [37–42]. Agents like carboplatin, cisplatin, 5-FU, cyclophosphamide, doxorubicin (or epirubicin), vincristine plus or minus prednisone, and etoposide were used with the hope to improve significantly patients outcome. In fact etoposide was better tolerated and showed a significant response in one study [43]. Unfortunately, still to date there is no first-line chemotherapy established for Merkel cell carcinoma patients. In fact, chemotherapy is used either in advanced-stage disease or in patients with recurrent, nonresectable, or disseminated disease. Therefore, the outcome is very controversially discussed in the literature. In particular, in a retrospective analysis including a huge number of patients' adjuvant chemotherapy was linked to a worse overall survival compared to patients who did not received chemotherapy [44]. 

Without doubt new systemic therapeutic strategies are needed for patients with Merkel cell carcinomas. One of such new strategies is termed as targeted anticancer therapies. Such therapies are shown to be very promising options in treating different types of cancer, that is, gastrointestinal tumors [45] or renal cell carcinomas [46]. Due to the rareness of the disease, a very limited number of studies are available. The first studies showed that c-kit, a receptor tyrosine kinase, is in 15–90% expressed by Merkel cell carcinoma cells. Recently, we conducted a study looking at a distinct panel of target proteins and we could find that therapeutically useful targets c-kit, Bmi-1, Mcl-1, VEGF-A and VEGF-C, VEGF-R2, PDGF-**α** and PDGF-**β** were expressed in Merkel cell carcinoma [9]. Another recently published study showed that survivin was a promising candidate for a new target therapy in Merkel cell carcinoma [47]. Looking at these studies the results are very promising and validate further clinical studies on the use of multitargeted tyrosine kinase inhibitors and antisense oligonucleotides in Merkel cell carcinoma [9].

Recently two studies showed that targeting MCPyV can be a promising option in patients with Merkel cell carcinoma [26, 27].

## 4. Imaging

For patients with Merkel cell carcinomas, imaging and subsequently staging of the tumor are of utmost importance. Since the introduction of ultrasonography in the late seventies, sonography of the neck is a key staging tool for patients with Merkel cell carcinoma. First reports on sonography and Merkel cell carcinoma were published in the late 90s [48]. Beyond ultrasonography, CT and MRI scanning are important for determining tumor size, location, and eventual bone invasion [48, 49]. In the late nineties, octreotide scanning in Merkel cell carcinoma patients was proposed to show a reliable detecting rate compared to CT and MRI imaging [50]. In the following years, however, it was shown that the octreotide scan has a low sensitivity and specificity [50]. Another whole body imaging technique, FDG-PET and PET-CT scanning showed highly reliable and accurate images in Merkel cell carcinoma patients with metastatic disease [49]. 

Sentinel node biopsy was introduced by Cabanas in 1977 in patients with penile carcinoma [51] enabling detection of micrometastasis in lymph nodes. This technique gains more and more importance in the management of patients with Merkel cell carcinoma since studies showed that patients with negative neck nodes have a risk of 30% to harbor micrometastasis in the neck nodes [52]. Another significant benefit of sentinel node imaging and mapping is an option to avoid the morbidity of an elective neck dissection in sentinel node negative patients [52–58].

## 5. Perspectives

Since the discovery of the Merkel cell in the skin in the 19th century and the description of the Merkel cell carcinoma in the early 70s, many new implementations in medicine with regard to diagnosis, imaging, and treatment have been introduced.

However, the management of patients with Merkel cell carcinoma is a tremendous challenge for the clinician as well as the patient and their families. The first step for optimal treatment is clinical investigation and proper diagnostic work-up of the patient including determination of the histology, either by excision biopsy or fine needle biopsy, imaging of the tumor and any metastatic disease, and finally determination of the therapeutic plan within a multidisciplinary setting.

In particular, diagnosis of Merkel cell carcinoma is based upon the CK20 positivity determined by immunohistochemistry whereas staging relies on ultrasonography, sentinel node, and CT/MRI and PET-CT scanning. Primary treatments including surgical resection and radiotherapy are currently the treatment of choice. In patients with recurrent either locoregional or distant metastasis, treatment options are very limited. In the case of resectable locoregional disease, surgical resection is an accurate way of treatment and for most of the patients it is unfortunately the only therapeutic option. However, in the presence of distant metastatic disease, there are no established systemic therapeutic regimens. The number of studies focusing on the development of new targeted anticancer therapy is steadily rising, and thus there is hope that new drug regimes for patients with distant and systemic Merkel cell carcinoma disease will be available in the near future. In particular, many study groups are looking for new strategies to target the Merkel cell polyoma virus either to prevent infection or to inhibit viral-induced carcinogenesis.
